# Association Between *STAT4* rs7574865 Polymorphism and Rheumatoid Arthritis: Debate Unresolved

**DOI:** 10.2174/1874312901812010172

**Published:** 2018-10-24

**Authors:** Iman Tarakji, Wafa Habbal, Fawza Monem

**Affiliations:** 1 Department of Biochemistry and Microbiology, Faculty of Pharmacy, Damascus University, Damascus, Syria; 2 Clinical Laboratories Department, Al-Assad Hospital, Damascus University, P.O. Box 10769, Damascus, Syria

**Keywords:** *STAT4* rs7574865, Rheumatoid arthritis, Syria, ACPA, SNP, MHC

## Abstract

**Background:**

*STAT4* rs7574865 polymorphism has been evidently associated with susceptibility to Rheumatoid Arthritis (RA) in European and Eastern Asian populations, whereas studies in other countries reported otherwise.

**Objective:**

We investigated the distribution of *STAT4* rs7574865 polymorphism in a group of Syrian RA patients.

**Methods:**

Eighty-one RA patients and forty healthy controls were enrolled and *STAT4* rs7574865 was genotyped by direct sequencing. RA patients were stratified according to Anti-Citrullinated Protein Antibodies (ACPA) status for analysis.

**Results:**

Minor T allele frequencies were 30.4%, 16.7%, and 23.8% in ACPA-positive RA patients, ACPA-negative RA patients, and healthy controls, respectively. No significant differences in *STAT4* rs7574865 allele/genotype frequencies were found between ACPA-positive RA patients, ACPA-negative RA patients, and healthy controls (P>0.05).

**Conclusion:**

*STAT4* rs7574865 TT genotype showed a potential impact on ACPA positivity in Syrian RA patients. However, *STAT4* rs7574865 effect on RA onset and severity is minor compared to other genetic factors such as *HLA-DRB1* shared epitope alleles.

## 1 INTRODUCTION

Rheumatoid Arthritis (RA) is one of the most common chronic multifactorial autoimmune disorders [1]. It gives rise to synovial joint deformity and loss of function leading to disabilities and early mortalities [2]. Genetic background plays a great role, side by side with environmental factors, in RA pathogenesis, etiology, prognosis and outcomes [3]. *HLA-DRB1* shared epitope alleles remain the major contributor to RA susceptibility [4]. Their involvement has been proved to be restricted to Anti-Citrullinated Protein Antibodies (ACPA)-positive patients [5]. Beyond the Major Histocompatibility Complex (MHC) region, many Single Nucleotide Polymorphisms (SNPs) occurring within numerous genes, including *STAT4* [6, 7], have been identified as candidate genetic markers associated with RA [5].

Signal Transducer and Activator of Transcription 4 (*STAT4*) gene encodes for a transcription factor that lies in the signaling pathway of IL-12 and IL-23 leading to the production of IFNγ and differentiation of CD4^+^ T-cells into Th_1_ and Th_17_ [8, 9]. The pathophysiological hallmark of RA involves the exaggeration of Th_1_/Th_17_ mediated inflammatory response [8] and upregulation of *STAT4* gene in synovial macrophages [10]. A susceptibility haplotype formed of four SNPs in *STAT4* gene and tagged by the T allele of rs7574865 has been shown to have a significant association with RA [11] evidently in Europe and Eastern Asia [6, 8, 11-19]. However, studies in other countries reported otherwise [20, 21], which suggests the need for ethnic-specific association studies. In this preliminary study, we aimed at investigating the distribution of *STAT4* rs7574865 polymorphism for the first time among a group of Syrian RA patients adding to the data that have only been scarcely collected in the Middle East.

## 2 MATERIALS AND METHODS

This case-control study included 81 unrelated RA Syrian patients presenting at outpatient clinics of Ibn Al-Nafis Hospital, Ministry of Health or admitted to the departments of arthrology at Al-Mowasah and Al-Assad Hospitals, Damascus University, between January 2010 and September 2011. All patients were diagnosed by an accredited arthrologist and they fulfilled the American College of Rheumatology (ACR) 1987 revised criteria [22]. Patients with juvenile idiopathic arthritis or other autoimmune diseases were excluded. Patients' clinical data including disease duration, Erythrocyte Sedimentation Rate (ESR), C-Reactive Protein (CRP), Rheumatoid Factor (RF), and ACPA were obtained from their medical records.

Forty healthy unrelated controls matched for age and ethnicity were also enrolled in the study. Neither controls nor any of their first degree relatives had RA or any other autoimmune disease. An informed consent was obtained from both patients and healthy individuals whose *HLA-DRB1* genotype has been analyzed previously [23]. This study has been approved by the Research Ethics Committee of Damascus University.

DNAs extracted from whole blood samples were genotyped for *STAT4* rs7574865 by direct DNA sequencing. A 182 bp-fragment was amplified using a forward primer 5'-GGT GTG GAT GGA GGT AAG GA-3' and a reverse primer 5'-ATC CCC TGA AAT TCC ACT GA-3' [24] manufactured by VBC Biotech Service (Vienna, Austria). 25-µL PCRs, including 2.5 µL DNA and 0.5 µM of each primer, were performed using a HotStar PCR SuperMix kit (GeneDirex, Las Vegas, NV) on the MasterCycler^®^ Pro S (Eppendorf, Hamburg, Germany). Thermal cycling was initiated at 94°C for 2 min, followed by 45 cycles of denaturation at 94°C for 2 min, annealing at 55°C for 30 sec and extension at 72°C for 2 min, and a final extension at 72°C for 7 min. Agarose gel electrophoresis (2.5%) was used for DNA visualization.

PCR products were purified using a High Pure PCR Product Purification Kit (Roche Diagnostics, Mannheim, Germany) and sequenced using the forward primer and a BigDye^®^ Terminator v3.1 Cycle Sequencing Kit on the ABI PRISM^®^ 3100-*Avant^TM^* Genetic Analyzer (Applied Biosystems, Foster City, CA) according to the manufacturers' instructions. *STAT4* rs7574865 genotypes GG, GT and TT were considered as wild-type, heterozygote mutant, and homozygote mutant, respectively.

Samples were analyzed for Hardy-Weinberg Equilibrium (HWE) using a chi-square goodness-of-fit test of SNPStats [25]. Differences of allele/genotype distribution of both *STAT4* rs7574865 and *HLA-DRB1* among study groups were analyzed using Kruskal-Wallis H and Mann-Whitney U tests. Whenever a significant difference was inferred, Kendall's tau-b correlation coefficient and Odds Ratio (OR) were calculated to test the strength and direction of the association between relevant variables. Statistical analyses were performed using IBM SPSS Statistics 19.0 software (International Business Machines Crop., New York, USA) and MedCalc for Windows, version 17.7.1 (MedCalc Software, Ostend, Belgium) and P-value <0.05 was considered significant.

## 3 RESULTS

Our study included 65 (80.25%) females and 16 (19.75%) males aged 41.4±10.6 years, and whose RA disease lasted for 11.3±6.3 years with ESR and CRP values of 56.7±29.7 mm/hr and 31.1±38.4 mg/L, respectively. In addition, 55 of 81 (67.90%) RA patients were positive for RF and 51 of 81 (62.96%) RA patients were positive for ACPA with values of 160.7±92.4 RU/mL. On the other hand, healthy controls included 26 (65%) females and 14 (35%) males aged 39.42 ±10.90 years.

The frequencies of presumably Risky (R) *HLA-DRB1* alleles (DRB*01, *04, and *10) were 59 of 102 (57.8%), 20 of 60 (33.3%), and 16 of 80 (20%), while the frequencies of presumably protective (P) *HLA-DRB1* alleles (DRB*11 and *13) were 17 of 102 (16.7%), 16 of 60 (26.7%), and 34 of 80 (42.5%) in ACPA-positive RA patients, ACPA-negative RA patients, and healthy controls, respectively Table **1**.

The frequencies of *STAT4* rs7574865 G allele were 71 of 102 (69.6%), 50 of 60 (83.3%), and 61 of 80 (76.3%), while the frequencies of *STAT4* rs7574865 T allele were 31 of 102 (30.4%), 10 of 60 (16.7%), and 19 of 80 (23.8%) in ACPA-positive RA patients, ACPA-negative RA patients, and healthy controls, respectively Table **2**.

The frequencies of *STAT4* rs7574865 GG genotype (wild-type) were 26 of 51 (51%), 20 of 30 (66.7%), and 25 of 40 (62.5%), the frequencies of *STAT4* rs7574865 GT genotype (heterozygote mutant) were 19 of 51 (37.3%), 10 of 30 (33.3%), and 11 of 40 (27.5%), and the frequencies of *STAT4* rs7574865 TT genotype (homozygote mutant) were 6 of 51 (11.8%), 0 of 30 (0%), and 4 of 40 (10%) in ACPA-positive RA patients, ACPA-negative RA patients, and healthy controls, respectively Table **2**.

No deviation from Hardy-Weinberg equilibrium was found in the control group (P=0.18). *HLA-DRB1* allele/genotype frequencies were significantly different between ACPA-positive RA patients, ACPA-negative RA patients, and healthy controls (P=0.00), where R allele was weakly correlated with ACPA positivity (τ=0.38, P=0.00) and P allele was weakly correlated with healthy status (τ=0.33, P=0.00). Moreover, ACPA positivity was 6.8 and 4.7 times more likely to occur in patients with R allele compared with healthy controls (OR 6.8, 95% CI:2.7-17.5, P=0.0001) and ACPA-negative patients (OR 4.7, 95% CI:1.7-12.7, P=0.002), respectively. In addition, healthy status was 4.5 and 7.2 times more likely to be maintained in individuals with P allele compared with ACPA-negative patients (OR 4.5, 95% CI:1.6-12.5, P=0.004) and ACPA-positive patients (OR 7.2, 95% CI:2.8-18.4, P<0.0001), respectively. When compared in pairs in terms of *HLA-DRB1* alleles, R alleles were not significantly different between ACPA-negative RA patients and healthy controls (P=0.21) and P alleles were not significantly different between ACPA-positive RA patients and ACPA-negative RA patients (P=0.24) Table **1**.

No significant differences in *STAT4* rs7574865 allele/genotype frequencies were found between ACPA-positive RA patients, ACPA-negative RA patients, and healthy controls (P>0.05). When compared in pairs in terms of *STAT4* rs7574865 genotype, homozygote mutant and wild-type genotypes were solely significantly different between ACPA-positive RA patients and ACPA-negative RA patients (P=0.041), where ACPA-positivity was not necessarily associated with TT genotype (OR 10.06, 95% CI:0.5-189, P=0.123) albeit weakly correlated (τ=0.278, P=0.045) Table **2**. However, no significant correlation was found between *STAT4* rs7574865 and *HLA-DRB1* alleles/genotypes in ACPA-positive RA patients, ACPA-negative RA patients, and healthy controls (P>0.05).

## 4 DISCUSSION

The Minor Allele Frequency (MAF) of *STAT4* rs7574865 among RA patients in Syria (25.3%) seems consistent with Tunisians (25%) [26] and Europeans (24%-30%) [6, 8, 12-14, 17, 27, 28] but higher than African Americans (15%) [20] and lower than Iranians (50%) [29], Eastern Asians (35%-42%) [11, 15, 16, 19], and Colombians (38%) [18]. On the contrary, the MAF among healthy controls in Syria (23.8%) seems higher than Tunisians (17%) [26] and Europeans (17%-23%) [6, 8, 12-14, 17, 27, 28] and still higher than African Americans (13%) [20] and lower than Iranians (50%) [29], Eastern Asians (31%-33%) [11, 15, 16, 19], and Colombians (31%) [18]. Regarding the neighboring countries, the MAF of *STAT4* rs7574865 has only been reported in Egypt. Albeit diverse among both RA patients (8% and 37%) and healthy controls (5% and 17%), Egyptian MAFs were inconsistent with those of our study [30, 31].

While the MAFs among ACPA-positive patients (30.4%; 60%) were perceptibly higher versus ACPA-negative patients (16.7%; 39.4%) in Syria and Iran [29], respectively, they appeared comparable in the European [8, 13, 14] and Eastern Asian [15] populations. Moreover, the TT genotype among ACPA-negative patients was exclusively nil in Syria and Egypt [31], a phenomenon unseen in other populations such as Turkey [21], Iran [29], Europe [13], and Eastern Asia [16, 19].

In discord with more than twenty studies conducted in Europe [6, 8, 12-14, 17, 27, 28], Eastern Asia [10, 11, 15, 16, 19], Colombia [18], Tunisia [26], and Egypt [30, 31], the minor T allele of *STAT4* rs7574865 was not associated with RA in Syria (p>.05) as well as in Turkish [21], Iranian [29], and African American [20] populations. This might be interpreted by the ethnicity impact which is underlined by intra-population consistency and inter-population inconsistency perceived in these studies. In addition, the average odds of having RA was only 1.5 (1.15-1.9) times higher in the presence of the minor T allele, which does not indicate a considerable effect despite the reported significant association in certain populations.

On the other hand, the reports on the effect of TT genotype showed vast disparity. While no effect was evidenced in seven studies [10, 13, 16, 18, 29, 30] including ours, the average odds of having RA was 1.5, 4.9, and 8.4 higher in five European and Eastern Asian [11, 13, 15, 27], one Egyptian [31], and one Tunisian [26] studies, respectively. Although our study does not prove the effect of TT genotype on having RA, our data suggest a potential effect of TT genotype on ACPA status; our RA patients with TT genotype were more likely to be ACPA-positive indicating a higher severity.

Being located in the third intron of *STAT4* gene, rs7574865 SNP biological impact is still debated. Whereas it does not disrupt any transcription factor binding site [28], alternative splicing mechanism was suggested and *STAT4* gene upregulation was established [32]. Collectively, functional and statistical studies hitherto refer to the small effect size of *STAT4* rs7574865 SNP regarding both RA onset and severity. The remarkable discrepancies among different populations in terms of minor T allele frequency, TT genotype frequency, presence/absence of association with RA status, and strength of association denote the higher impact of other genetic and environmental factors. *HLA-DRB1* alleles/genotypes, for instance, were weakly-to-moderately correlated with RA onset and ACPA status. In addition, the odds of having RA with positive ACPA was as high as 7 times in the presence of an *HLA-DRB1* shared epitope allele (DRB*01, *04, and *10).

The reported average onset of RA usually ranges between the ages of 30 and 60; hence case-control studies with young adult healthy controls might get biased. Furthermore, the larger the sample size the higher the statistical power. However, the presence/absence of an association between minor T allele and RA does not seem relevant to the sample size and age as much as the ethnicity of subjects enrolled in the published studies [26, 29-31]. Moreover, the inference of our statistical analyses remained unchanged when confined to healthy controls over 40 years of age (data not shown).

## CONCLUSION

Our preliminary study points to a potential impact of *STAT4* rs7574865 TT genotype on ACPA positivity and a more significant impact of *HLA-DRB1* shared epitope alleles on RA onset and ACPA status. However, we failed to pinpoint an association between *STAT4* rs7574865 minor T allele and RA susceptibility. More studies are recommended to be conducted repeatedly in different ethnic groups so that the debate on such an association might be resolved. Being involved in the RA-associated inflammatory response, *STAT4* gene might exhibit other variations to be investigated alike in future studies.

## Figures and Tables

**Table 1:** Distribution of *HLA-DRB1* alleles/genotypes among Syrian rheumatoid arthritis patients and healthy controls.

	**RA Patients** **n (%)**			**Controls** **n (%)**	**Statistical Analyses**					
	**Total** **(n=81)**	**ACPA+** **(n=51)**	**ACPA-** **(n=30)**	**(n=40)**	**Patients** ***vs*** **controls**	**ACPA+ pts** ***vs*** **controls**	**ACPA- pts** ***vs*** **controls**	**ACPA+ pts** ***vs*** **ACPA- pts** ***vs*** **controls**	**ACPA+ pts** ***vs*** **ACPA- pts**	**ACPA+ GG/TT pts** ***vs*** **ACPA- GG/TT pts**
**HLA-DRB1 *alleles***										
**Subjects with R allele(s)**	55 (67.9%)	41 (80.4%)	14 (46.7%)	15 (37.5%)	p=.002 (MWW) τ=.269, p=.003 OR=3.5, p=.002	p=.00 (MWW) τ=.438, p=.00 OR=6.8, p=.0001	p=.444 (MWW)	p=.00 (K-W) τ=.364, p=.00	p=.002 (MWW) τ=.349, p=.002 OR=4.7, p=.002	p=.005 (MWW) τ=.392, p=.005
**Subjects with P allele(s)**	27 (33.3%)	15 (29.4%)	12 (40%)	30 (75%)	p=.00 (MWW) τ=.281, p=.002 OR=6, p<.0001	p=.00 (MWW) τ=.453, p=.00 OR=7.2, p<.0001	p=.003 (MWW) τ=.354, p=.003 OR=4.5, p=.004	p=.00 (K-W) τ=.362, p=.00	p=.332 (MWW)	p=.793 (MWW)
**R alleles frequency**	79 (48.7%)	59 (57.8%)	20 (33.3%)	16 (20%)	p=.00 (MWW) τ=.309, p=.00	p=.00 (MWW) τ=.474, p=.00	p=.208 (MWW)	p=.00 (K-W) τ=.383, p=.00	p=.007 (MWW) τ=.286, p=.007	p=.011 (MWW) τ=.331, p=.011
**P alleles frequency**	33 (20.4%)	17 (16.7%)	16 (26.7%)	34 (42.5%)	p=.00 (MWW) τ=.278, p=.002	p=.00 (MWW) τ=.429, p=.00	p=.023 (MWW) τ=.264, p=.023	p=.00 (K-W) τ=.333, p=.00	p=.242 (MWW)	p=.667 (MWW)
**HLA-DRB1 *genotypes***										
**RR**	24 (29.6%)	18 (35.3%)	6 (20%)	1 (2.5%)	p=.001 (MWW) τ=.239, p=.004	p=.00 (MWW) τ=.350, p=.00	p=.061 (MWW)	p=.002 (K-W) τ=.246, p=.001	p=.644 (MWW)	p=.978 (MWW)
**RN**	21 (25.9%)	15 (29.4%)	6 (20%)	4 (10%)						
**RP**	10 (12.3%)	8 (15.7%)	2 (6.7%)	10 (25%)						
**NN**	9 (11.1%)	3 (5.9%)	6 (20%)	5 (12.5%)						
**PN**	11 (13.6%)	5 (9.8%)	6 (20%)	16 (40%)						
**PP**	6 (7.4%)	2 (3.9%)	4 (13.3%)	4 (10%)						

RA, Rheumatoid Arthritis; ACPA, Anti-Citrullinated Protein Antibodies; pts, RA patients; R, Risky *HLA-DRB1* allele (DRB1*01, 04, 10); P, protective *HLA-DRB1* allele (DRB1*11, 13); N, non-risky, non-protective *HLA-DRB1* allele; GG/TT, *STAT4* rs7574865 homozygous genotype; MWW, Mann–Whitney U test; K-W, Kruskal-Wallis test; τ, Kendall's tau coefficient; OR, Odds Ratio.

**Table 2:** Distribution of *STAT4* rs7574865 alleles/genotypes among Syrian rheumatoid arthritis patients and healthy controls.

	**RA Patients** **n (%)**			**Controls** **n (%)**	**Statistical Analyses**					
	**Total** **(n=81)**	**ACPA+** **(n=51)**	**ACPA-** **(n=30)**	**(n=40)**	**Patients** ***vs*** **controls**	**ACPA+ pts** ***vs*** **controls**	**ACPA- pts** ***vs*** **controls**	**ACPA+ pts** ***vs*** **ACPA- pts** ***vs*** **controls**	**ACPA+ pts** ***vs*** **ACPA- pts**	**ACPA+ GG/TT pts** ***vs*** **ACPA- GG/TT pts**
**STAT4 *rs7574865 alleles***										
**Subjects with G allele(s)**	75 (92.6%)	45 (88.2%)	30 (100%)	36 (90%)	p=.628 (MWW)	p=.790 (MWW)	p=.077 (MWW)	p=.161 (K-W)	p=.052 (MWW)	p=.045 (MWW) τ=.278, p=.045
**Subjects with T allele(s)**	35 (43.2%)	25 (49%)	10 (33.3%)	15 (37.5%)	p=.550 (MWW)	p=.274 (MWW)	p=.721 (MWW)	p=.323 (K-W)	p=.171 (MWW)	p=.029 (MWW) τ=.304, p=.029
**G allele frequency**	121 (74.7%)	71 (69.6%)	50 (83.3%)	61 (76.3%)	p=.670 (MWW)	p=.311 (MWW)	p=.525 (MWW)	p=.233 (K-W)	p=.095 (MWW)	p=.029 (MWW) τ=.301, p=.029
**T allele frequency**	41 (25.3%)	31 (30.4%)	10 (16.7%)	19 (23.8%)	p=.670 (MWW)	p=.311 (MWW)	p=.525 (MWW)	p=.233 (K-W)	p=.095 (MWW)	p=.029 (MWW) τ=.301, p=.029
**STAT4 *rs7574865 genotypes***										
**GG**	46 (56.8%)	26 (51%)	20 (66.7%)	25 (62.5%)	p=.754 (MWW)	p=.392 (MWW)	p=.525 (MWW)	p=.310 (K-W)	p=.131 (MWW)	p=.045 (MWW) τ=.278, p=.045
**GT**	29 (35.8%)	19 (37.3%)	10 (33.3%)	11 (27.5%)						
**TT**	6 (7.4%)	6 (11.8%)	0 (0%)	4 (10%)						

RA, Rheumatoid Arthritis; ACPA, Anti-citrullinated Protein Antibodies; pts, RA patients; *STAT4*, signal transducer and activator of transcription 4 gene; MWW, Mann–Whitney U test; K-W, Kruskal-Wallis test; τ, Kendall's tau coefficient.
